# Inhibition of BRD4 enhanced the tumor suppression effect of dasatinib in gastric cancer

**DOI:** 10.1007/s12032-022-01831-8

**Published:** 2022-11-09

**Authors:** Hao Shen, Xuefei Hu, Xinrui Yang, Jiahui Chen, Yating Fu, Hongwei He, Yongkang Shi, Rong Zeng, Wenjun Chang, Shangyong Zheng

**Affiliations:** 1 Department of Navy Environmental and Occupational Health, Faculty of Naval Medicine, Navy Military Medical University, Shanghai, People’s Republic of China; 2 School of Medicine, Yunnan University, Kunming, Yunnan People’s Republic of China; 3 Department of Medical Oncology, The First People’s Hospital of Yunnan Province, The Affiliated Hospital of Kunming University of Science and Technology, Kunming, Yunnan People’s Republic of China

**Keywords:** Gastric cancer, BRD4, Molibresib, Dasatinib, Prognosis, Targeted therapy

## Abstract

BRD4, a member of the bromodomain and extraterminal (BET) family, is elevated in multiple cancer tissues, including gastric cancer (GC). Targeted therapy with BRD4 may help improve the overall survival of patients with GC. Meanwhile, the approved multi-target kinase inhibitor, dasatinib, was recently reported to show varied tumor-suppressive effects in GC cells. This study investigated BRD4 expression in vivo and in vitro using immunohistochemistry and western blotting, respectively. We discussed the relationship between BRD4 expression and patient prognosis. Next, the antitumor efficacy of dasatinib was measured in BRD4-knockdown GC cells to determine the role of BRD4 blockage in dasatinib treatment. Finally, molibresib, a BET inhibitor, was used to measure the cooperative function of BRD4 inhibition and dasatinib treatment in three GC cell lines. Epithelial BRD4 expression was higher in tumoral and metastatic tissues and was strongly associated with unfavorable tumor, node, and metastasis stages and survival. BRD4 expression was heterogeneous in the three GC cell lines tested in vitro. In SGC7901, a BRD4-high GC cell line, knockdown of BRD4 using specific siRNAs suppressed cell growth individually and cooperatively with dasatinib. Moreover, molibresib and dasatinib showed a cooperative effect in suppressing the proliferation of BRD4-high GC cells. In conclusion, we confirmed that increased epithelial BRD4 expression is associated with poor disease stage and prognosis in GC and BRD4 blockage might be a valuable strategy to improve the sensitivity of dasatinib and other drugs in the chemotherapy of advanced GC.

## Introduction

Gastric cancer (GC) is one of the most frequently occurring cancers worldwide, with more than 26,000 new cases and 11,000 estimated deaths in America in 2021 [1]. Most early-stage GC patients have a satisfactory prognosis following surgery, while advanced-stage GC patients exhibit a poor prognosis with surgery alone. For non-resectable metastatic GC in particular, treatment options are limited to chemotherapy. Although perioperative or adjuvant chemotherapy with infusions of 5-fluorouracil, leucovorin, oxaliplatin, and docetaxel could significantly improve the prognosis of those patients [2, 3], the development of novel therapeutic strategies for advanced GC is still necessary.

Targeted cancer therapy has already been used for multiple cancers; however, the high molecular and phenotypic heterogeneity of GC greatly limits the application of targeted therapies. For example, trastuzumab, the human epidermal growth factor 2 (HER2) inhibitor, which is the first licensed targeted agent for GC, shows restricted benefits owing to the low amplification rate (17–20%) of HER2 in GC patients [4]. Therefore, identifying general molecular targets in patients with GC is imperative. BRD4, a member of the bromodomain and extraterminal (BET) family, plays transcriptional and epigenetic regulatory roles in embryogenesis, apoptosis, and inflammatory response, particularly in cancer development [5–7]. Several inhibitors targeting BET bromodomains have been developed and employed in clinical studies. These inhibitors not only directly attenuate tumor cell growth but also enhance antitumor activity when co-administered with conventional treatment regimens, such as cisplatin [8] and HER2 inhibitor (lapatinib) [9]. In GC, the expression of BRD4 is significantly higher in tumor tissues than in adjacent non-tumor tissues [6]. Inhibition of BRD4 has been reported to suppress GC progression and metastasis by destabilizing snail family transcriptional repressor 1 (SNAIL) or downregulating c-myc expression [5, 6]. BET bromodomain inhibitors, such as iBET-151, ARV-825, and JQ1, are employed in GC in vivo and in vitro and demonstrate effective antitumor activity [10–12]. Therefore, BRD4 is a promising therapeutic target for GC treatment. Another BRD4 inhibitor, molibresib, which binds the tandem bromodomains of BET with high affinity, has already been employed in phase I and phase II clinical trials of various solid tumors, such as ovarian cancer [13], colorectal cancer (NCT03266159), and other advanced malignant solid neoplasms (NCT03925428). However, its application in GC has never been studied.

Dasatinib, an oral multi-target kinase inhibitor approved for leukemia treatment, targets multiple cancer kinases, including BCR-ABL and SRC families, exhibits strong antitumor activity and weak side effects. Dasatinib has also shown efficacy in GC, as SRC is constitutively overexpressed in most GC tissues. Shi et al. reported that dasatinib targets *p*-SRC to sensitize the anti-proliferation and migration function of oxaliplatin in GC [14]. Meanwhile, Choi et al. showed that dasatinib has varied efficacy in GC cell lines by inhibiting the novel target p90-RSK [15]. However, the function of the combination of BRD4 inhibition and dasatinib treatment on GC has not been reported.

In the present study, we analyzed the association between BRD4 expression, clinical features, and patient survival in a GC cohort (*n* = 550). We studied the effect of BRD4 blockage combined with dasatinib treatment in GC in vitro. The results showed that the expression of BRD4 was elevated in GC and its high expression in tumor tissues was shown to indicate poor prognosis in GC patients. The inhibition of BRD4 by siRNAs and molibresib facilitated the sensitivity of GC cells to dasatinib. Our results implied that BRD4 played a crucial role in GC prognosis and may be a promising therapeutic target for improving the chemosensitivity of dasatinib.

## Materials and methods

### Cell lines and cell culture

Human GC cell lines (AGS, SGC7901, and HGC-27) were purchased from the Cell Bank of the Chinese Academy of Sciences (Shanghai, China). The cells were maintained in DMEM medium supplemented with 10% fetal bovine serum (FBS) and 2% penicillin/streptomycin (Bio-Channel) in a 5% CO_2_ incubator.

### Primers and real-time PCR

Primers for human BRD4 and GAPDH were as follows (5′ to 3′): BRD4-forward: CTGACTAGTGTCGACATGTCTGCGGAGAGC and BRD4-reverse: GATATCCTCGAGTCAGAAAAGATTTTCTTC; GAPDH-forward: TGCACCACCAACTGCTTAGC; and GAPDH-reverse: GGCATGGACTGTGGTCATGAG. Real-time PCR was carried out using a LightCycler 480 II system (Roche) with SYBR qPCR Master Mix (Vanzyme), followed by pre-denaturation at 95 °C for 30 s, amplification at 95 °C for 30 s, and 60 °C for 10 s, for 40 cycles. The relative mRNA levels of each gene were calculated using the 2^−△△Ct^ value.

### Antibodies and western blot

Anti-human BRD4 (1:800, ab128874) and anti-human GAPDH (1:5000, ab9485) antibodies were purchased from Abcam. For western blot experiments, protein extracts were separated by SDS-PAGE and transferred onto 0.45 μm PVDF membranes (Millipore). Membranes were then blocked in PBST containing 5% non-fat milk and hybridized overnight by incubating with the primary antibody at 4 °C and subsequent incubation for 1 h with the secondary antibody at room temperature. Membranes were developed using Luminata™ Forte Western HRP substrate (Millipore) and exposed in Amersham Imager 600 (GE).

### Small interfering RNAs (siRNAs) and gene knockdown

Two pairs of siRNAs targeting the consensus sequences of BRD4 transcript variants and a control siRNA were designed (siBRD4-1: GCACAGCUAGCUGAAGAGAdTdT; siBRD4-2: AAGACCCUUGUGCUCGUUGUCdTdT; siControl: UUCUCCGAACGUGUCACGUTT) and synthesized by Shanghai Invitrogen. The siRNAs were transfected into GC cells at a final concentration of 20 nM with the Lipofectamine RNAi MAX reagent (Invitrogen).

### Specimens and immunohistochemistry

Formalin-fixed, paraffin-embedded (FFPE) samples of 43 para-cancerous, 518 cancerous, and 22 metastatic tissues were obtained from 550 GC patients who underwent surgical resection at the First People’s Hospital of Yunnan Province from January 2006 to November 2011. Patients who had received preoperative chemotherapy or radiation were excluded from the study. The primary outcomes of interest were disease-specific survival (DSS) and disease-free survival (DFS). DSS was defined as the number of months from the surgery date to the date the patient died of GC. DFS was defined as the number of months from the surgery date to the date of the first relapse. This study was approved by the institutional review committee of the First People's Hospital of Yunnan Province. All participants signed a written informed consent form, providing permission for the biomaterials to be used in the study. Tissue microarrays (TMAs) containing FFPE specimens were constructed and used to study the expression pattern of BRD4 by immunohistochemistry (IHC). Briefly, slides were first baked at 85 °C for 10 min, deparaffinized in xylene, and rehydrated in a graded ethanol series. Antigens were retrieved by placing all slides in boiling sodium citrate (10 mmol/L, pH 6.0, 100 °C) for 30 min after blocking for 5 min with 3% H_2_O_2_ to remove endogenous peroxidase. Slides were incubated overnight at 4 °C with rabbit anti-human BRD4 polyclonal antibody (1:1000, HPA059180; Sigma-Aldrich), according to the manufacturer’s instructions. After incubation for 30 min with secondary antibodies from the Elivision TM super HRP (Mouse/Rabbit) IHC Kit (Maxvision), the slides were reacted in diaminobenzidine (DAB) solution for 45 s and stained with hematoxylin for 25 s. All slides were stained simultaneously by the same researcher to eliminate intra-assay variation.

### Cell proliferation assay

Cells were plated in triplicate at 5000 cells/well in 96-well plates. Cell viability was assayed 24 to 72 h after treatment with dasatinib or transfection with BRD4 siRNAs and siControl. For the drug combination experiments, serial dilutions of dasatinib and molibresib were added to the cultured GC cells for 2 days. Finally, the cell population was measured using the Cell Counting Kit-8 (Dojindo) according to the manufacturer’s instructions. Absorbance at 450 nm was measured to determine the viable cell population. The survival rate (SR) and synergy score of the GC cells after treatment were calculated using R software version 4.2.1 (packages: Bioconductor, https://www.bioconductor.org/). Primary screening was performed with SGC7901 cells, and the identified hits were further tested with AGS and HGC-27 cells.

### Colony formation assay

Cells were resuspended and seeded into six-well plates (Corning) at a density of 1000–1500 cells/well. Dasatinib was added, and siBRD4 or siControl was transfected every 2 days for each group. After 7–14 days, the supernatant was removed, and the cell colonies were stained with crystal violet solution. The assay was performed in triplicate. The plates were scanned using a photo-scanner, and cell growth was quantified using ImageJ software.

### Quantification and statistical analysis

To compare BRD4 expression between GC and adjacent normal tissues, we used independent sample *T* tests for nonpaired samples and paired *T* tests for paired samples. Categorical data, such as gender and tumor differentiation grade, were compared and analyzed using the chi-square or Mann–Whitney *U* test. For survival analysis, the patient subgroups, which were divided using SPSS according to BRD4 protein levels, were compared using the Kaplan–Meier method and univariate and multivariate Cox proportional hazards models. The log-rank test was used to assess the statistical significance of the Kaplan–Meier curves. All statistical tests were two-sided and performed using IBM SPSS statistics 20 for Windows. Statistical significance was set at *p* < 0.05.

## Results

### BRD4 was elevated in GC in vivo and in vitro

**Fig. 1:**
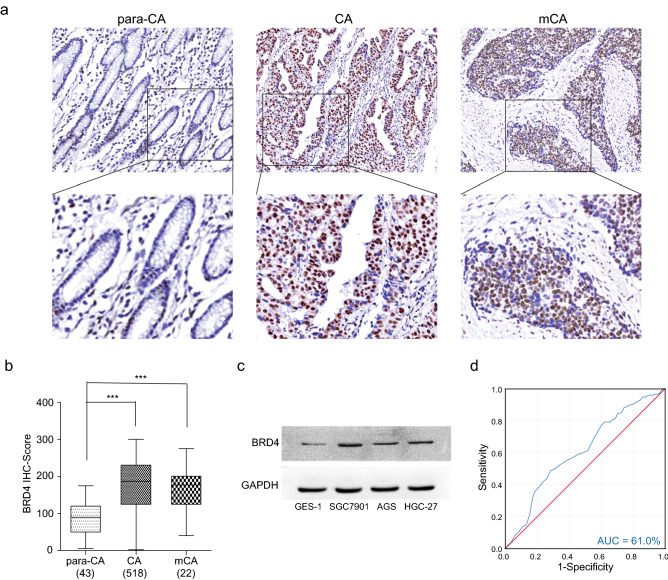
BRD4 was elevated in GC in vivo and in vitro. **a** Immunohistochemistry staining of BRD4 in gastric tissues. **b** H-score of BRD4 in each group. **c** Expression of BRD4 in normal gastric cells (GES-1) and GC cells (SGC7901, AGS, HGC-27). **d** ROC curves analysis of BRD4 expression and GC patients outcome in our cohort. *para-CA* para-cancer tissue, *CA* cancerous tissue, *m-CA* metastatic cancer tissue; ***p < 0.001

To investigate the relationship between BRD4 and GC, we firstly examined the expression pattern of BRD4 in GC specimens from our cohort by IHC staining. The results showed that BRD4 mainly located in the nuclei of epithelial cells in gastric tissues. Further analysis showed that the expression of BRD4 was elevated nearly twofold in epithelial cells in tumoral and metastasis regions compared with normal regions, which implied a tumor-promoting function of BRD4 in GC (Fig. 1a, b). We then examined the expression of BRD4 in vitro. The protein levels of BRD4 in the normal gastric epithelial cell line GES-1 and three GC cell lines (SGC7901, AGS, and HGC-27) were measured by western blotting. BRD4 was also clearly elevated in GC cells and extremely high in SGC7901 cells (Fig. 1c). These results showed the enhanced expression of BRD4 in GC revealed its promoting function in gastric oncogenesis and pointed out the possibility of antitumor therapy targeting BRD4 in GC. Next, we performed ROC curves analysis for BRD4 expression and patient outcome, the area under curve (AUC) was 61% in our cohort, which gave evidence for BRD4 to serve as a prognosis marker for GC (Fig. 1d).

### Epithelial BRD4 expression was associated with the differentiation grade and TNM stage of GC

ROC curve was used to identify the optimal cut-off value, and patients in the cohort were divided into BRD4-high (*H*-score > 212.5, *N* = 337) and BRD4-low (*H*-score ≤ 212.5, *N* = 181) subgroups. Pearson chi-square test (for categorical variables) or Mann–Whitney *U* test (non-parametric) was used to analyze the relationship between epithelial BRD4 expression and patient clinical features. As shown in Table 1, significant associations were identified between epithelial BRD4 expression and differentiation grade (*p* = 0.015) or TNM stages (*p* < 0.001), whereas there was no relationship among BRD4 expression and other clinical features of GC patients. These results revealed that epithelial BRD4 might be a predictive factor for the tumorigenesis and progression of GC.

**Table 1:** Characteristics of patients in the GC cohort dichotomized by BRD4 expression

Variables	brd4^Low^ (*n* = 337)	brd4^High^ (*n* = 181)	*p*-value
Sex, *n* (%)			
Male	240 (71.2)	131 (72.4)	0.835
Female	97 (28.8)	50 (27.6)	
Differential grade, *n* (%)			
Well + Moderate	228 (67.7)	103 (56.9)	**0.015**
Poor	109 (32.3)	78 (43.1)	
TNM stage, *n* (%)			
I	113 (35.3)	53 (29.3)	** < 0.001**
II	90 (26.7)	48 (26.5)	
III + IV	128 (38)	80 (44.2)	
Chemotherapy, *n* (%)			
Yes	227 (67.4)	122 (67.4)	0.992
No	110 (32.6)	59 (32.6)	
Serum CEA, *n* (%)			
< 5 ng/mL	246 (73)	143 (79)	0.068
≥ 5 ng/mL	91 (27)	38 (21)	
Serum CA199, *n* (%)			
< 37 U/mL	255 (75.7)	139 (76.8)	0.639
≥ 37 U/mL	79 (23.4)	42 (23.2)	
Missing	3 (0.9)		

Bold values indicate significance of p value (*p* < 0.05)

CEA carcinoembryonic antigen, CA199 carbohydrate antigen 199

### High epithelial BRD4 expression predicted unfavorable prognosis in GC

**Fig. 2:**
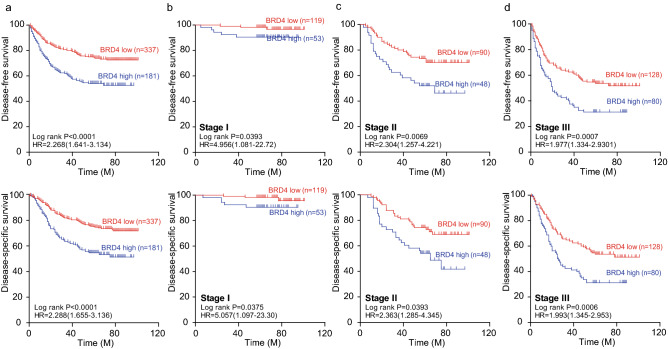
High epithelial BRD4 expression predicted unfavorable prognosis in GC. **a** Associations between BRD4 expression and the overall DFS or DSS of GC patients. **b–d** Patients were divided into subgroups via TNM stages. The associations between BRD4 expression and DFS or DSS in each subgroup

To further identify the prognostic role of epithelial BRD4 expression in GC, Kaplan–Meier analysis was used to show the contribution of BRD4 expression to disease-free survival (DFS) and disease-specific survival (DSS). The patients were divided into BRD4-high and BRD4-low groups based on the cut-off value (212.5). The results showed remarkable differences between BRD4-high and BRD4-low patients: high epithelial BRD4 expression predicted significantly shortened DFS and DSS (Fig. 2a). We then examined the prognostic value of epithelial BRD4 at different TNM stages. For all three stages, high BRD4 expression was associated with poorer DFS and DSS, and the significance increased from stage I to stage III, which indicated a prognostic function of epithelial BRD4 expression in GC (Fig. 2b–d). Next, Cox regression analysis was used to further investigate the associations among BRD4 expression, clinical features of patients and survival. Results of Univariate Cox regression analysis showed that BRD4 expression, differential grade, chemotherapy conditions, and TNM stage were significantly associated with DFS and DSS of GC patients. Further multivariate Cox analyses showed that, except for chemotherapy conditions, the other three features were independent risk factors for GC patients, in which high BRD4 indicated poor prognosis with an HR of 2.228 (95% CI 1.642–3.023; *p* < 0.001) for DFS and an HR of 2.232 (95% CI 1.645–3.028; *p* < 0.001) for DSS (Table 2).

**Table 2:** Cox regression analysis of epithelial BRD4 expression and clinicopathological covariates in the GC cohort

	DFS				DSS				
	Univariate analysis		Multivariate analysis		Univariate analysis		Multivariate analysis		
Variables	HR (95% CI)	*p*-value	HR (95% CI)	*p*-value	HR (95% CI)	*p*-value		HR (95% CI)	*p*-value
Brd4									
Low vs High	2.087 (1.560–2.835)	** < 0.001**	2.228 (1.642–3.023)	** < 0.001**	2.103 (1.560–2.835)	** < 0.001**		2.232 (1.645–3.028)	** < 0.001**
Differential grade									
(Well + Moderate) vs Poor	0.701 (0.507–0.970)	**0.032**	0.715 (0.512–0.997)	**0.048**	0.701 (0.506–0.970)	**0.032**		0.714 (0.512–0.997)	**0.048**
Chemotherapy									
Yes vs No	0.358 (0.240–0.534)	** < 0.001**	0.850 (0.553–1.306)	0.458	0.358 (0.240–0.534)	** < 0.001**		0.845 (0.550–1.299)	0.443
TNM stage									
(I + II) vs III	2.833 (2.307–3.478)	** < 0.001**	2.769 (2.204–3.480)	** < 0.001**	2.838 (2.309–3.487)	** < 0.001**		2.762 (2.198–3.470)	** < 0.001**
Age									
≤ 60 vs > 60	0.954 (0.706–1.289)	0.759			0.945 (0.699–1.276)	0.711			
Sex									
Male vs Female	0.781 (0.552–1.105)	0.163			0.775 (0.547–1.097)	0.150			
Serum CEA (ng/mL)									
< 5 vs ≥ 5	1.020 (0.693–1.502)	0.919			1.020 (0.693–1.501)	0.920			
Serum CA199 (U/mL)									
< 37 vs ≥ 37	0.905 (0.571–1.435)	0.672			0.910 (0.574–1.442)	0.678			

*HR* hazard ratio, *CI* confidence interval, *CA199* carbohydrate antigen 199, *CEA* carcinoembryonic antigen

### BRD4 knockdown enhanced the growth suppression of dasatinib on GC cells

**Fig. 3:**
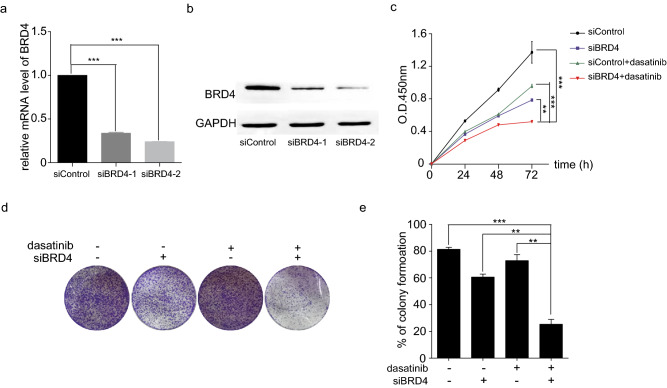
BRD4 knockdown enhanced the growth suppression of dasatinib on GC cells. **a **and** b** Knockdown efficiency of BRD4 by siRNAs. **c** CCK-8 assay of SGC7901 cells been treated with siRNAs, dasatinib, or their combination. **d** and **e** Colony formation assay of SGC7901 cells treated with siRNAs, dasatinib, or their combination. **p < 0.01; ***p < 0.001

To further investigate the tumor promotion effect of BRD4 in GC, BRD4 was knocked down by siRNAs interference in SGC7901 cells. RT-qPCR and western blotting results showed that the expression of BRD4 effectively decreased at the transcriptional and protein levels by 60% and 80%, respectively (Fig. 3a, b). CCK-8 and colony formation assays were performed to detect the proliferation and growth of BRD4-compromised GC cells. As expected, SGC7901 cells transfected with siBRD4 showed remarkable reduced cell proliferation and colony formation (Fig. 3c–e). We then compared the growth inhibition efficiency of BRD4 knockdown and dasatinib treatment in GC cells. Surprisingly, siBRD4-transfection showed comparative growth inhibition efficiency with dasatinib treatment in GC cells. Moreover, BRD4 blockage combined with dasatinib treatment resulted in the strongest growth inhibition in both CCK-8 and colony formation assays (Fig. 3c–e), which revealed a sensitization effect of BRD4 knockdown on GC cell growth inhibition induced by dasatinib.

### Molibresib and dasatinib cooperatively suppressed the proliferation of GC cells

**Fig. 4:**
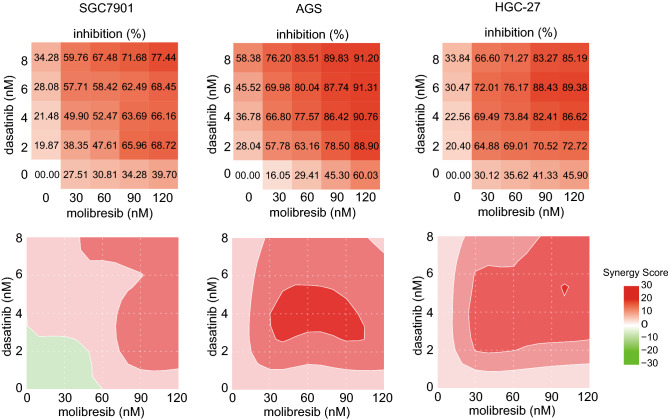
Molibresib and dasatinib showed synergistic anti-tumor function on GC cells. Inhibitory effect and synergy score of GC cells been treated with molibresib, dasatinib, or their combination

Molibresib is considered the most promising BRD4 inhibitor and has already been used in phase II clinical trials. Since BRD4 knockdown showed a growth-suppressive effect in GC cells alone or in combination with dasatinib, we speculated that molibresib could also suppress the proliferation of GC cells and may showed synergistic effect with dasatinib. The CCK-8 assay was conducted to test the proliferation of three GC cell lines treated with molibresib, dasatinib, and their combination. As shown in Fig. 4, molibresib enhanced the growth suppression effect of dasatinib and combined treatment with 60–90 nM molibresib and 2–4 nM dasatinib exhibited synergistic effects in all three cell lines. These results revealed that molibresib, a BRD4 inhibitor, and dasatinib, a multi-target kinase-inhibiting chemotherapeutic drug, synergistically suppressed GC cell proliferation in vitro.

## Discussion

GC is the most common cancer among digestive system neoplasms [16]. As GC develops insidiously and metastasizes easily, patients are usually diagnosed at an advanced stage. The frequently used clinical tumor markers for digestive system cancer, such as serum CEA, CA199, and CA125, have proven unreliable because of their low positive rate in early-stage GC.

First-line chemotherapy for patients with inoperable metastatic GC mainly consists of platinum and fluoropyrimidine. Still, the mortality rate of these patients remains high, with a median survival of less than 12 months [4]. To solve this problem, targeted therapies have been licensed to treat GC. Trastuzumab, an anti-HER2 monoclonal antibody, has been used separately or cooperatively as first-line targeted therapy for patients with advanced GC. 5-fluorouracil-based chemotherapy with trastuzumab can reduce postoperative recurrence in GC patients and prolong their survival [17, 18]. However, the application of trastuzumab is strongly limited by the low HER2-positive rate in patients with GC (< 20%). It is of great significance to identify new and effective biomarkers for the early diagnosis and treatment of GC.

This study consisted of 43 normal, 22 metastatic, and 518 primary tumor specimens from patients with GC from China. Based on this cohort, we compared the translational levels of BRD4 between tumor and non-tumor tissues. Regardless of the primary tumor and metastasis, the IHC score confirmed a remarkable increase of BRD4 expression in tumor tissues. We also measured the expression of BRD4 in vitro. There was only a slight expression of BRD4 in GES-1, a normal gastric epithelial cell line, while the three GC cell lines exhibited inconsistently high expression of BRD4. This result indicated the importance and heterogeneity of BRD4 in GC. We also investigated the association between BRD4 expression and various clinical features. On the one hand, elevated BRD4 expression was related to the differentiation grade and TNM stages, suggesting that BRD4 may be a potential marker candidate for the malignant grade of GC, especially in early cases. On the other hand, a high BRD4 IHC score was associated with shortened DFS and DSS in patients with GC. We also showed such a relationship in separate TNM stages, from stage I to stage III, and the remarkable differences between BRD4-low and BRD4-high patients increased gradually. This result indicated the prognostic function of epithelial BRD4 in both early and advanced GC.

Targeting BRD4 by inhibiting BET bromodomains has been employed in animal models and clinical studies. It is especially looked forward to improving the chemotherapeutic effect of advanced malignant solid neoplasms. However, the effects of BRD4 inhibitors in clinical trials for multiple cancers are unsatisfactory. This may be caused by some therapeutic limitations, such as off-target effects, drug resistance, and the multifactorial nature of most cancers. As a result, combined chemotherapies or dual-target inhibitors based on BRD4 and other kinases inhibition, such as PI3K/BRD4 dual inhibitors, have been exploited with increasing intensity [19, 20]. We focused on dasatinib, an approved multi-target kinase inhibitor with varied tumor-suppressive effects in GC cells and further studied the antitumor function of BRD4 inhibition in combination with dasatinib treatment in GC cells.

When we employed BRD4 knockdown in one of the BRD4-high cell lines SGC7901, a clear suppression of tumor growth was observed in BRD4-compromised cells, which implied a direct antitumor function of BRD4 blockage. When the BRD4-compromised cells were treated with low-dose dasatinib, and their proliferation and colony formation were further inhibited. We carried out dasatinib and molibresib, a BRD4 inhibitor, alone or combined in GC cells to confirm this result. As expected, molibresib and dasatinib exhibited significant synergetic functions in the three BRD4-high GC cell lines, which indicated the sensitization effect of molibresib in dasatinib therapy.

In summary, we systematically described the expression pattern and clinical significance of BRD4 in GC and provided evidence that BRD4 may be a valuable prevention and prognostic biomarker in GC. Blockage of BRD4 reduced the growth of GC in vitro, which supporting the tumor-promoting role of BRD4 in this type of cancer. Molibresib, a targeted drug with a high affinity to bromodomains of BRD4, showed favorable antitumor activity in various GC cells, and the inhibition of BRD4 activity by siRNAs or molibresib could facilitate the chemosensitivity of dasatinib in GC cells. Further employment of this combination regimen of molibresib and dasatinib in xenograft mice model would provide evidence for its application in clinical use.
